# The impact of *Bacillus thuringiensis* var.* israelensis* (Vectobac^®^ WDG) larvicide sprayed with drones on the bio-control of malaria vectors in rice fields of sub-urban Kigali, Rwanda

**DOI:** 10.1186/s12936-024-05104-9

**Published:** 2024-09-17

**Authors:** Dunia Munyakanage, Elias Niyituma, Alphonse Mutabazi, Xavier Misago, Clarisse Musanabaganwa, Eric Remera, Eric Rutayisire, Mamy Muziga Ingabire, Silas Majambere, Aimable Mbituyumuremyi, Mathew Piero Ngugi, Elizabeth Kokwaro, Emmanuel Hakizimana, Claude Mambo Muvunyi

**Affiliations:** 1 Malaria and Other Parasitic Diseases Division of Rwanda Biomedical Center, Ministry of Health, Kigali, Rwanda; 2 Charis Unmanned Aerial Solutions Ltd, Kigali, Rwanda; 3 Valent BioSciences Corporation, Illinois, USA; 4 Department of Zoological Sciences, Kenyatta University, Nairobi, Kenya

**Keywords:** Malaria, Mosquitoes, Drones, Rice fields, *Bacillus thuringiensis*, Rwanda

## Abstract

**Background:**

The core vector control tools used to reduce malaria prevalence are currently long-lasting insecticidal nets (LLINs), and indoor residual spraying (IRS). These interventions are hindered by insecticide resistance and behavioural adaptation by malaria vectors. Thus, for effective interruption of malaria transmission, there is a need to develop novel vector control interventions and technologies to address the above challenges. Larviciding using drones was experimented as an innovative tool that could complement existing indoor interventions to control malaria.

**Methods:**

A non-randomized larviciding trial was carried out in irrigated rice fields in sub-urban Kigali, Rwanda. Potential mosquito larval habitats in study sites were mapped and subsequently sprayed using multirotor drones. Application of *Bacillus thuringiensis* var. *israelensis* (Bti) (Vectobac^®^ WDG) was followed by entomological surveys that were performed every two weeks over a ten-month period. Sampling of mosquito larvae was done with dippers while adult mosquitoes were collected using CDC miniature light traps (CDC-LT) and pyrethrum spraying collection (PSC) methods. Malaria cases were routinely monitored through community health workers in villages surrounding the study sites.

**Results:**

The abundance of all-species mosquito larvae, *Anopheles* larvae and all-species pupae declined by 68.1%, 74.6% and 99.6%, respectively. Larval density was reduced by 93.3% for total larvae, 95.3% for the *Anopheles* larvae and 61.9% for pupae. The total adult mosquitoes and *Anopheles gambiae *sensu lato collected using CDC-Light trap declined by 60.6% and 80% respectively. Malaria incidence also declined significantly between intervention and control sites (U = 20, z = − 2.268, p = 0.023).

**Conclusions:**

The larviciding using drone technology implemented in Rwanda demonstrated a substantial reduction in abundance and density of mosquito larvae and, concomitant decline in adult mosquito populations and malaria incidences in villages contingent to the treatment sites. The scaling up of larval source management (LSM) has to be integrated in malaria programmes in targeted areas of malaria transmission in order to enhance the gains in malaria control.

## Background

Mosquitoes are well-established vectors of *Plasmodium* parasites, accountable for spreading malaria. Female *Anopheles* mosquitoes seek human blood and in the process act as carriers and transmitters of disease pathogens [1]. Malaria remains a deleterious public health problem in subtropical and tropical countries [2] with more than two billion people exposed to it. Globally and according to 2023 World malaria report, 249 million cases of malaria have been reported in 2022 with 608 000 deaths. The African region is the most affected with an estimation of 94% and 95% of malaria cases and deaths, respectively. In the same region, children under 5 years of age accounted for about 80% of all reported malaria deaths [3]. A wide range of *Anopheles* mosquitoes have been incriminated in malaria transmission in the African region, mainly belonging to the *Anopheles gambiae* and *Anopheles funestus* species complexes [4].

In Rwanda, an increase in malaria morbidity from 208,000 malaria cases in 2011 to 4,637,483 cases in 2016 was reported [5]. This upsurge in malaria cases was observed in all provinces of Rwanda [5]. Despite the efforts deployed to reverse the curve, malaria still is a public health burden in Rwanda and its intensity is not uniform in all districts [6]. The persistence of malaria cases suggests that the vectors utilize the residual breeding habitats during dry season and subsequently maintain malaria transmission [7]; thus, this poses a challenge in designing vector control interventions.

The current malaria control measures in Rwanda have primary focused on the control of indoor adult mosquitoes. The measures include use of long-lasting insecticidal nets (LLINs) and indoor residual spraying (IRS), preventing the disease by limiting human-vector contact. The widespread use of IRS and LLINs have not been sufficient to eliminate malaria as standalone vector control tools, mainly due to insecticide resistance, outdoor and residual transmission [8]. Thus, there is an actual need for supplemental vector control tools to effectively tackle existing limitations [9]. The larval source management (LSM) is one of the oldest malaria control intervention extensively deployed to control mosquitoes at the source [10] It was broadly used in earlier malaria elimination programmes, and currently as a primary vector control intervention to prevent the reintroduction of eliminated mosquito-borne diseases including malaria [11]. Currently, LSM is recommended by the World Health Organization (WHO) for controlling mosquito breeding habitats that are “few, fixed and findable” in high endemic areas [12].

Different field trials performed in malaria endemic countries of Africa and Asia regions demonstrated drastic reductions of mosquito density and abundance, mosquito infection rates, as well as the prevalence of malaria infections [13]. However, other field trials revealed that LSM may not work in complex rural larval habitats, for instance, in extensive flooding ecosystems [14]. Thus, the successful LSM requires taking prior account of vectors species and their ecology in planning of interventions. In 2014 and for the first time, Rwanda experimented larviciding using *Bacillus thuringiensis* var. *israelensis* (Bti) in rural rice field habitats. The hand applications using knapsack sprayers proved limitations to ensure a full coverage of all identified breeding sites. That trial recommended the exploration of aerial spraying methods to improve the coverage in complex mosquito breeding habitats [15].

There is dearth of data on the bio-ecology of malaria vectors in the study district of Gasabo District, in peri-urban of Kigali City. The supplemental vector-based control interventions such as larval source management (LSM) using drones for prior mapping mosquito breeding water bodies and then for spraying of *Bti*, involving a community participation would potentially be suitable to sustainably overcome the hand based application challenges [16]. The aim of the present study was to evaluate the key entomological parameters (abundance and density of mosquito populations) and epidemiological trends of malaria incidence at community following *Bti* application using drone based technology for prior mapping of breeding sites and treatment with bio-larvicide. Thus, novel LSM applications techniques that use drones and community engagement in the application of LSM may usually become more appealing as breeding sites are focalized mainly in the man-made breeding sites such as the targeted agriculture-based mosquito habitats.

## Methods

### Study site

This study was conducted in Gasabo District which occupies the northern half of Kigali City approximately 9.8Kms from Kigali city with fifteen administrative sectors. It has an area of 430.30 km^2^ of which a big portion (84%) is rural while the small portion (16%) represents the developed urban area (16%), with a population of 879,505 residents in 2022 with 81.2% of its population residing in urban areas [17]. The elevation is about 1456 m above sea level. Rainfall is generally bimodal, March to May is marked with long rains, and short rains from September to November. The short dry season starts from December to February and long dry season start from June to mid-September [18]. The annual average rainfall is 927 mm and the temperature ranges from 17 °C to 28 °C. Most of the population in Gasabo District are employed in Agriculture (31%), Trade (17%), Government (11%) [19]. Malaria in Gasabo District is mesoendemic and the main vectors are *An. gambiae *sensu stricto (*s.s*.) and *Anopheles arabiensis*. Both the intervention and control arms sites benefited in February 2020, the universal distribution of insecticide-treated nets (ITNs), PermaNet^®^3.0, treated with the insecticide deltamethrin, and a synergist, piperonyl butoxide (PBO).

Rice farming is a major agricultural activity with two farming cycles throughout the year. The expected mosquito breeding sites are mainly made of the stagnant water in the rice fields, expected to be permanent for the first three months of the rice cultivation (January to March and July to September). Other potential mosquito breeding sites are mainly after rain season, include inter-crops water drains, pits and puddles from mining activities, water dams for harvesting rainwater for irrigation, stagnant water in the peri-domestic, water in different containers in use or unused.

### Study design

**Fig. 1:**
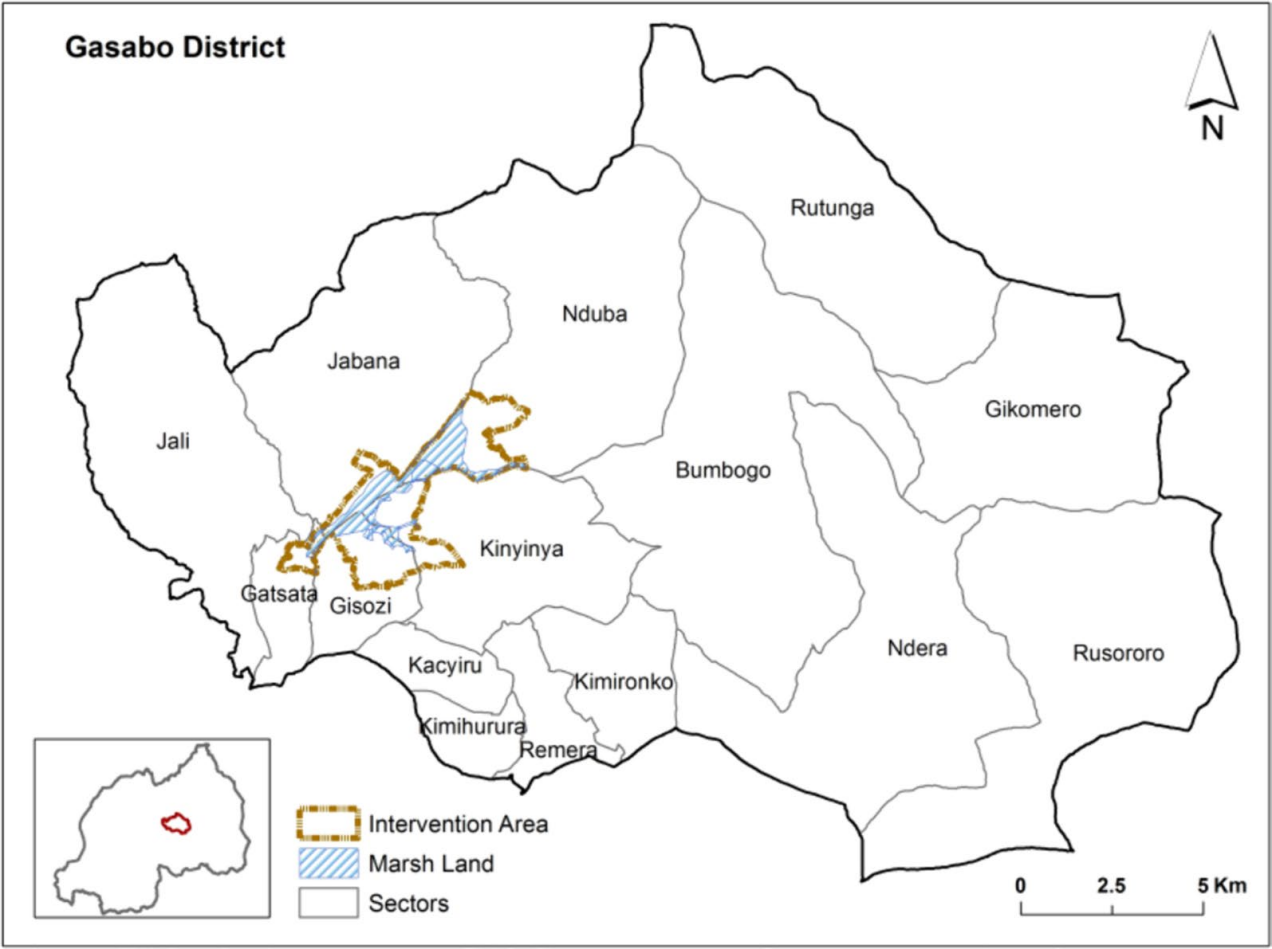
Location of the study site, Gasabo district, Kigali-City, Rwanda

**Fig. 2:**
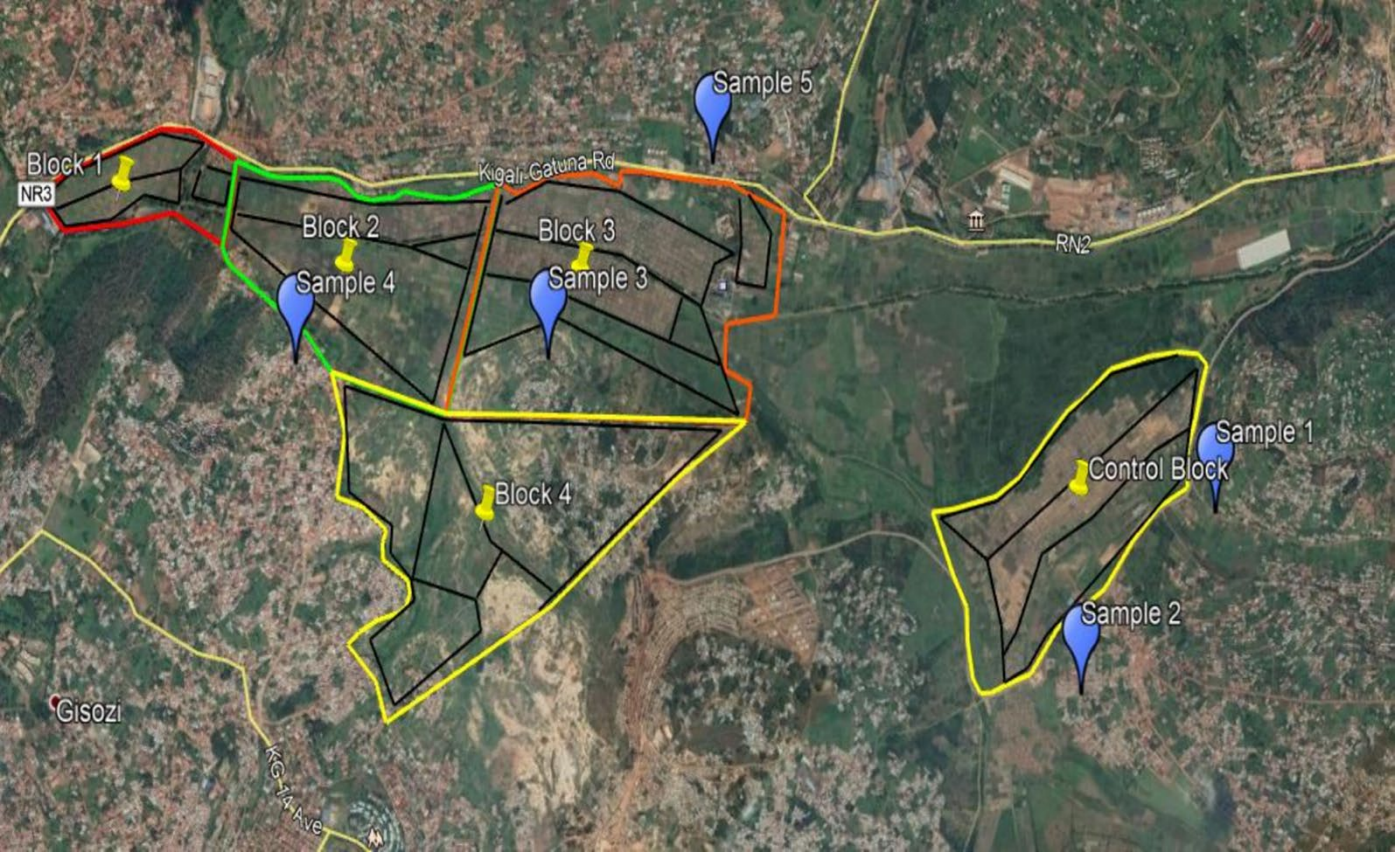
Aerial image of study site displaying experimental and control arms, intervention blocks and location of villages for adult mosquito sampling, Gasabo, Kigali-City, Rwanda

The study was non-randomized with control involving a total of five blocks of marshlands located into five sectors of Gasabo District. Four blocks of marshlands located in the sectors of Jabana, Gisozi, Gatsata, and Kinyinya, with a total area of 336 ha was the experimental arm and received *Bacillus thuringiensis* var. *israelensis (Bti)* application (Figs. 1, 2). The control arm was in the sector of Nduba with 78 ha and did not receive any larvicide application. the maps of all water bodies in the experimental and control parts were generated using drones, Model DJI Phantom 4RTK equipped with camera 20MP, 1-inch CMOS sensor, prior to the intervention and each time before *Bti* application.

The experimental sites were treated with Bti 3000 International Toxic Units (ITU) permg), of strain AM 65–52, traded under the name of VectoBac^®^ Water-Dispersible Granules (WDG). The product was supplied by Valent Biosciences Corporation, Illinois, USA. The active ingredient of the product is based on a mixture of free endotoxin protein crystals produced by Bti and the spores and cells bearing them [20]. It was applied every 2 weeks of intervals and using the Unmanned Aerial Vehicles (UAV) “drones”, Model Q16 with tank capacity of 17 L. Rice farmers were trained to spray *Bti* into the mosquito breeding sites not accessible by drones mainly the peri-domestic breeding sites and other areas identified as non-eligible for aerial spraying.

Considering the recommended dosage of *Bti* 3000 ITU/mg, strain AM 65–52, commercially traded as VectoBac^®^ WDG, 300 g were diluted in 10 L and covering one ha with aerial spraying with drone in 15 min, one drone was estimated to cover between 15 to 20 ha per day. For the supplemental hand application, sprayer pumps (agricultural knapsack sprayer pumps, Agriscope® model with tank capacity of 16 L), were calibrated for releasing 30 L of 300 g diluted *Bti* in one ha of water body.

### Larval density monitoring

**Fig. 3:**
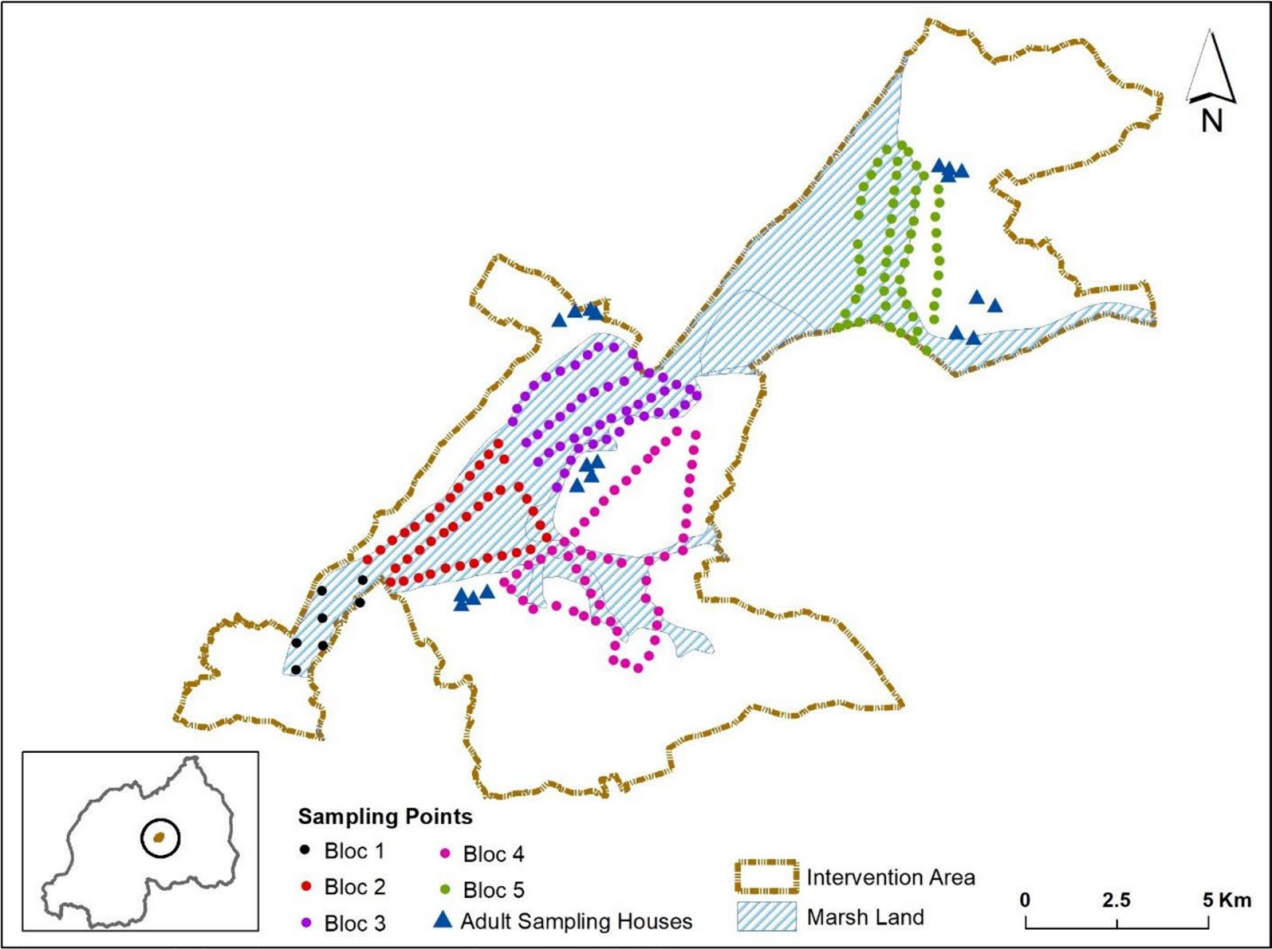
Sampling plots for mosquito larval stages and community houses for adult mosquito collections in the study site, Gasabo-Kigali-City, Rwanda. Total of 230 larval sampling plots, respectively split per bloc. A. Experimental site: Bloc 1, 51 plots, bloc 2, 35 plots, bloc 3, 60 plots, bloc 4, 35 plots. Control site: bloc 5, 49 plots

To measure the impact of larval control using *Bti*, baseline surveys on mosquito breeding habitats and larvae were carried out one week before the application of *Bti*, key entomological indicators were measured on larval densities. Larval sampling continued every two weeks for ten months, starting from two to three days post *Bti* spraying. The larval monitoring was performed in selected sampling plots purposively chosen using a Global Position System coordinate, sampling points marked at every 100 m alongside the marshlands in three line transects, middle and two ridges of the marshlands (Fig. 3). The overview of the numbers of sampling plots is as shown on Table 1.

**Table 1:** Results of abundance and density on aquatic stages and adult mosquitoes between treatment and control arms, calculated using generalized linear model (GLM), July 2020

Methods	Variable	Number of plots/houses	Mean (SE) Control	Mean (SE) Treatment	Mean Diff (SE)	P-Value
Dipping (larvae)	Anopheles larvae	131	3.53 (1.15)	3.01 (0.88)	0.52 (2.48)	> 0.05
	Culicines larvae	131	9.80 (2.41)	5.23 (1.92)	4.56 (5.43)	> 0.05
	Pupae	131	1.73 (0.55)	1.21 (0.34)	0.53 (0.97)	> 0.05
PSC	*Anopheles* sp.	40	0 (0)	0.54 (0.28)	− 0.54 (0.34)	> 0.05
	*Culicines* sp.	40	2.43(0.81)	1.54 (0.51)	0.895 (0.91)	> 0.05
CDC	*Anopheles* sp.	40	1.56 (0.46)	2.21 (0.62)	1.95 (0.41)	> 0.05
	*Culicines* sp.	40	34.06 (9.41)	13.25 (2.56)	21.57 (4.31)	< 0.05

The mean numbers were estimated: a) per dip for larvae or pupae, b) per house and per night for PSC method or per trap and per night for CDC-LT method for adult mosquitoes

Sampling was done using standard dippers (350 ml) by making five or ten dips (depending on the type of habitats) in each sampling plot. The presence was marked for plots found positive to mosquito larvae while negative was marked with plots without mosquito larvae. The results were recorded and categorized according to the mosquito species and their development stages.

### Mosquito adult sampling methods

Indoor mosquito collections were done for two successive nights using battery powered CDC miniature light traps and pyrethrum spray collection (PSC) to sample endophagic/endophilic vectors. The CDC-LT were installed at 1.5 m from the ground at the foot end of a bed net, occupied by a human sleeper [21] The PSC were performed early morning, from 5 to 7 am using pyrethrum insecticide mixed with piperonyl butoxide (PBO) as synergist. The spraying was performed inside houses and external walls, alongside the eaves. Knock down mosquitoes were collected on white sheets (2 × 2 m) placed on ground and then preserved in petri dishes per house, sampling date and intervention, for future identification and other advanced molecular tests [22]. Female adult mosquitoes were collected in twenty randomly selected houses from five different sites adjacent to the marshlands, three sites (12 houses) in neighbourhood of intervention area and two (8 houses) in control area (Fig. 3). Identification of mosquito species and sporozoite infection was done using morphological characteristics and PCR, and ELISA for detection of sporozoite infection.

The sampled adult mosquitoes were counted and identified using standard morphological features such as wing patterns, size, abdominal markings, meso pleural and thoracic hairs as described by Coetzee [23]. Each collected female anopheline mosquito was kept individually in a labelled micro-centrifuge tube with a lid for airtight locking with a desiccant.

Siblings of the collected female *An*. *gambiae *sensu lato (s.l.) were characterized using PCR technique from legs and wings mosquito parts [24]. To determine presence of a circumsporozoite proteins (CSP), ELISA tests was done on the head and thorax to detect *P. falciparum,* and the infection was confirmed through optical density (OD) measured using spectrophotometer [25]. Blood-meal sources of all collected blood-fed female samples collected by PSC method were analysed using a direct ELISA, using anti-host monoclonal antibodies (IgG) conjugate against human, cattle, goat, sheep, chicken proteins, and the OD was measured using spectrophotometer [26].

### The pre-intervention mosquito density and abundance

The abundance and density of anopheles, and culicines mosquito larvae and pupae in the study sampling plots were assessed before the intervention. This baseline density was also conducted on adult mosquitoes collected using CDC-LT and Pyrethrum Spraying Collection methods.

### Determination of malaria incidence

Community health workers (CHWs) patients’ registers were used for monthly data collection of malaria cases from the contingent villages to the study area. Fifteen villages in proximity to the marshlands of the study area, aggregated into twelve nearby the intervention and three in the control area, respectively.

### Statistical analysis

Data were recorded in Excel and transferred into statistical software, version R 4.0.2 for statistical analysis. In preliminary, descriptive analysis was performed to generate tables and curves. The intervention and control groups were convened to visualize differences in the responses between the two groups. Mean, median, and standard errors (SE) have been used to present continuous variables and frequencies for categorical variables.

A balance check was performed using the t-test to compare means of treatment and control groups for independent variables at baseline (Round 0). Further, the difference analysis was calculated using linear regression analysis, adjusted for time, to evaluate the effect of larviciding on anopheles and culicines mosquito larvae and pupae. The catches of adult mosquitoes fitted to a negative binomial distribution with log link function, and were analysed using Generalized Linear Model with time and type of treatments included as random variables. The differences in malaria incidence between intervention and control sites was determined using a non-parametric test, the Mann Whitney test.

### Ethical consideration

The study was presented to Rwanda Biomedical Centre, Division of Research, Innovation and Data Sciences for review and clearance and received approval Ref: No 225/RBC/2020. The importation and usage of *Bti* was authorized by the Ministry of Health, Department of Food and Drug Authority. Before application of *Bti* and larval monitoring, verbal consent was obtained from local leaders, the head of the rice farmer cooperative, owners of houses used for adult collections and the entomology technicians involved in mosquito monitoring.

## Results

### The pre-intervention mosquito abundance and density

The pre-intervention mosquito parameters were determined on mosquito larvae and pupae using 131 plots as well as on adult mosquitoes using 40 houses-nights. The results showed similarities between the treatment and control arms (Table 1).

### Larval habitat occupancy

**Fig. 4:**
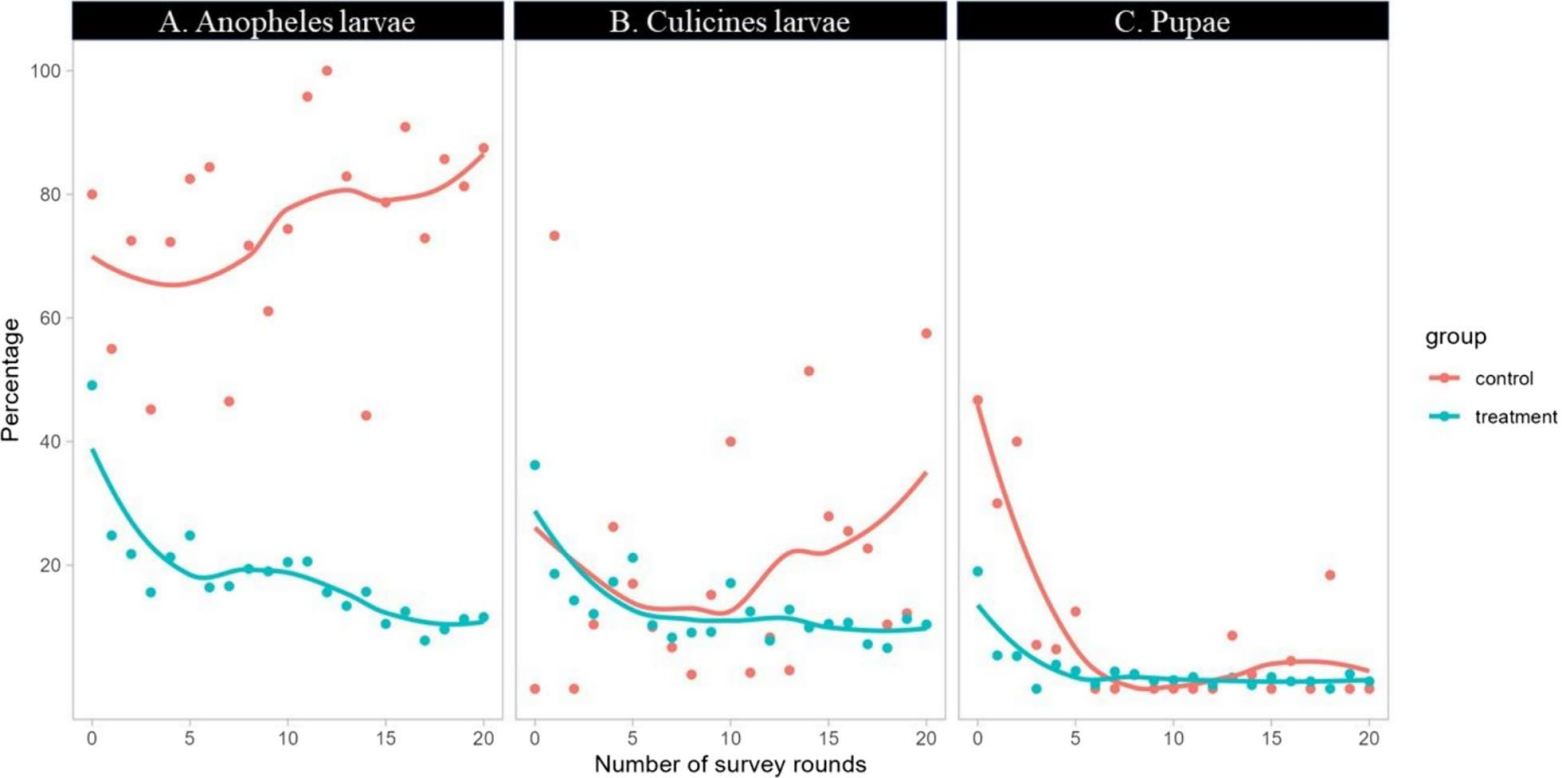
Trends of habitat occupancy per survey round in treatment and control arms for *Anopheles* larvae (**A**), culicines larvae (**B**) and pupae (**C**)

At the end of the intervention (Round 20), the comparison of treatment and control blocks showed a statistically significant between treatment and control blocks with a decline over time in the overall larval habitat occupancy (Fig. 4). In the experimental sites, the overall habitat occupancy of sampling plots was 16.1% for *Anopheles* larvae with a decrease of 78.3% (p < 0.001) while it was 74.3% in control. The overall habitat occupancy of sampling plots for Culicines larvae was 11.6% in experimental sites with a reduction of 31.4% while was 16.9% in control (p < 0.001), pupal occupancy rate was 1.9% with a reduction of 69.2% in experimental sites while was 6% in control (p < 0.001) (Table 2).

**Table 2:** Mean proportion habitat occupancy of late instars L3 + L4 of anopheles, culicines larvae and pupae in control and treatment arms during the 20 rounds of drones-based application of bio-larvicide (Bti), Rwanda

Mosquito larval species	Control arm (proportion of habitat occupancy in % (SE) [n = 832]	Treatment arm (proportion of habitat occupancy in %: n = 3196)	Difference % (SE)	P-Value
*Anopheles*	74.3 (1.5)	16.1 (0.6)	58.2 (1.5)	< 0.001*
*Culicines*	16.8 (1.3)	11.5 (0.6)	5.3 (1.3)	< 0.001*
*Pupae*	6.0 (0.8)	1.9 (0.2)	4.1 (0.7)	< 0.001*

The “*” explains the statistical differences at 95% of confidence interval

### Mosquito larval density

Analysis of larval density showed that in all blocks of the experimental study sites, the treatment intervention significantly reduced the anophelines larval density by 98.7%, culicines larval density by 81.3% and pupal density by 75% (Table 3). The *Anopheles* larval density declined and kept lower in treatment than control arms while a rebound was observed from 10th rounds in control for culicines and pupal stages (Fig. 4). This was due to the significant effects of time, treatment alone or in combination on results (Table 4).

**Table 3:** Mean number per dip (density) of late instars L3 + L4 of anopheles, culicines larvae and pupae in control and treatment arms during the 20 rounds of drones-based application of bio-larvicide (Bti), in Rwanda

Mosquito larval species	Control arm (Adjusted mean larvae per dip)	Treatment arm (Adjusted mean larvae per dip)	Reduction %	P-Value
*Anopheles*	0.67	0.01	98.7	< 0.001*
*Culicines*	0.08	0.02	81.3	< 0.001*
*Pupae*	0.02	0.01	75.0	< 0.001*

The “*” explains the statistical differences at 95% of confidence interval

**Table 4:** Effects of treatment and time on anopheles, culicines larvae and pupal stages

Mosquito species and stages	**Coefficient**	**[95% conf. Interval]**
Anopheles larval		
Treatment	− 0.25***	[− 0.32; − 0.18]
Time	− 0.43***	[− 0.48; − 0.37]
Time × Treatment	0.24***	[0.17;0.31]
Culicines larval		
Treatment	− 0.70***	[− 0.85; − 0.55]
Time	− 0.90***	[− 1.01; − 0.78]
Time × Treatment	0.70***	[0.55;0.86]
Pupa larval		
Treatment	− 0.22***	[− 0.25; − 0.19]
Time	− 0.24***	[− 0.26; − 0.22]
Time × Treatment	0.22***	[0.19;0.25]

### Adult mosquitoes

#### CDC-LT

Overall, out of 14,387 adult mosquitoes collected over ten months in the two study sites, culicines were dominant (85.9%, n = 12,354) and anophelines represented 14.1%. Among *Anopheles* mosquitoes collected, *An. gambiae s.l.* was the most abundant (90.3%, n = 1836) followed by *Anopheles ziemanni* (5.8%, n = 117), *Anopheles squamosus* (3%, n = 60), *Anopheles maculipalpis* (0.8%, n = 17), and other *Anopheles* mosquitoes (0.15%, n = 3). Out of the total mosquito collections, 74% (n = 1510) of *Anopheles* spp. and 55% (n = 6796) of Culicines spp were collected from the control arm (Table 5).

**Table 5:** Mean number of adult mosquitoes per trap and per night collected using CDC-Light trap in nearby villages of treatment (n = 480) and control arms (n = 320), during the 20 rounds of drones-based application of bio-larvicide (Bti), Rwanda

Mosquito species	Control arm: Mean + SE (n = 320)	Treatment arm: Mean + SE (n = 480)	Adjusted mean difference + SE	Reduction in %	P-Value
Tota*l Anopheles*	4.64 (0.34)	0.97 (0.06)	3.66 (0.296)	79.09	< 0.001*
*Culicines spp*	19.53 (1.03)	10.91 (0.43)	8.62 (0.99)	44.14	< 0.001*
*Anopheles gambiae s.l*	4.05 (0.30)	0.97 (0.06)	3.08 (0.258)	76.05	< 0.001*

The “*” explains the statistical differences at 95% of confidence interval

*Anopheles arabiensis* was found to be the predominant *An. gambiae* sibling species collected with CDC LT. For a total of 782 specimens of *An. gambiae s.l*. tested using species-specific PCR, 405 (51.8%) were identified as *An. arabiensis* and 377 (48.2%) as *An. gambiae s.s.*

ELISA assays to detect *P. falciparum* CSP protein were performed on the head and thorax of 2,108 specimens of individual female *Anopheles* mosquitoes collected by both CDC LT (79.6%, n = 1678) and PSC (20.4%, n = 430) methods to ascertain the malaria parasite infection rates in the study sites. *An. gambiae s.l*. (n = 1914) made up 90.8% of the total samples, *An. ziemanni* (5.4%, n = 114), *An. squamosus* (3.1%, n = 65), *An. maculipalpis* (0.5%, n = 10), *Anopheles rufipes* (0.1%, n = 3), *Anopheles coustani* (0.05%, n = 1), *Anopheles funestus* (0.05%, n = 1). All the samples tested negative for *Plasmodium* infection*.*

Larviciding using VectoBac^®^ WDG sprayed with drones showed a significant impact on adult mosquito densities in villages neighbouring the intervention sites (Table 5). The total adult *Anopheles* spp was significantly reduced by 79.9%, Culicines spp by 44.14% and *An. gambiae* s.l*.* by 76.05% (p < 0.001). The significant effects of treatment and time alone were observed on *Anopheles* mosquitoes, but not on Culicines. The combination of the two variables did not have any significant effect either on *Anopheles* nor on Culicines mosquitoes (Table 6).

**Table 6:** Effect of treatment and time alone or in combination on anopheles, culicines adult mosquitoes collected using CDC-LT method

	**Coefficient**	**[95% conf. Interval]**
Anopheles spp.		
Treatment	− 4.15***	(− 5.23; − 3.07)
Time	− 0.13***	(− 0.2; − 0.06)
Time × Treatment	0.07	(− 0.02;0.16)
*An. gambiae s.l*		
Treatment	− 3.51***	(− 4.45; − 2.57)
Time	− 0.12***	(− 0.18; − 0.06)
Time × Treatment	0.06	(− 0.02;0.14)
Culicines spp.		
Treatment	− 10.29*	(− 18.38; − 2.19)
Time	− 0.18	(− 0.43;0.08)
Time × Treatment	0.12	(− 0.21;0.45)

### Pyrethrum spraying collection method

**Fig. 5:**
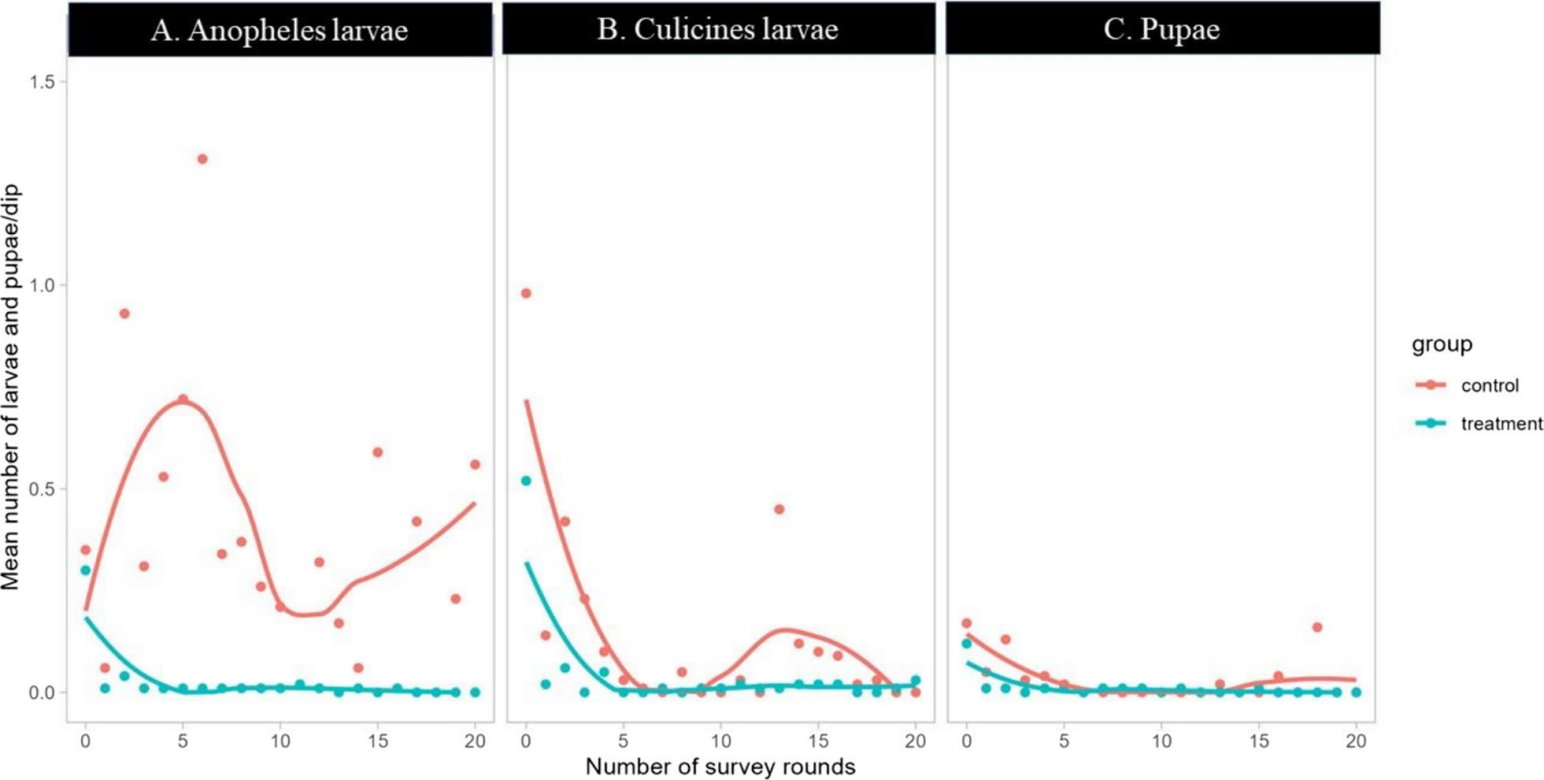
Trends of mosquito larval and pupal density per survey round in treatment and control arms for *Anopheles* larvae (**A**), culicines larvae (**B**) and pupae (**C**)

With PSC method, out of 1,839 adult mosquitoes collected over ten months in the two study sites, Culicines spp represented 79.4% (n = 1460) while total *Anopheles* mosquitoes were 21.6%. Among the catches of *Anopheles* mosquitoes, *An. gambiae s.l.* was the most dominant with 96.8% (n = 367) followed by *An. ziemanni* (2.9%, n = 11), *An. squamosus* (0.3%, n = 1). Per study arm, 73% (n = 379) of *Anopheles* spp. and 37% (n = 536) of Culicine mosquitoes were caught from the control arm (see Fig. 5).

*Anopheles arabiensis* was found to be the predominant *An. gambiae* sibling species collected. For a total of 362 specimens of An. *gambiae* s.l*.* with species-specific PCR results, 295 (81.5%) were successfully identified as *An. arabiensis* and 67 (18.5%) as *An. gambiae* s.s*.*

A total of 155 blood fed *An. gambiae* s.l*.* collected from the treatment and control houses were tested by direct ELISA for blood meal source identification. The majority (69.7%) of *Anopheles gambiae* s.l. had fed on bovine blood (n = 108), human IgG was detected in 12.3% (n = 19), remaining *An. gambiae* s.l. had fed on other vertebrate hosts, which were Goat (5.2%, n = 8), Bovine and Goat (3.9, n = 6), Human and Bovine (0.6, n = 1), and unidentified host in 8.4% (n = 13).

**Fig. 6:**
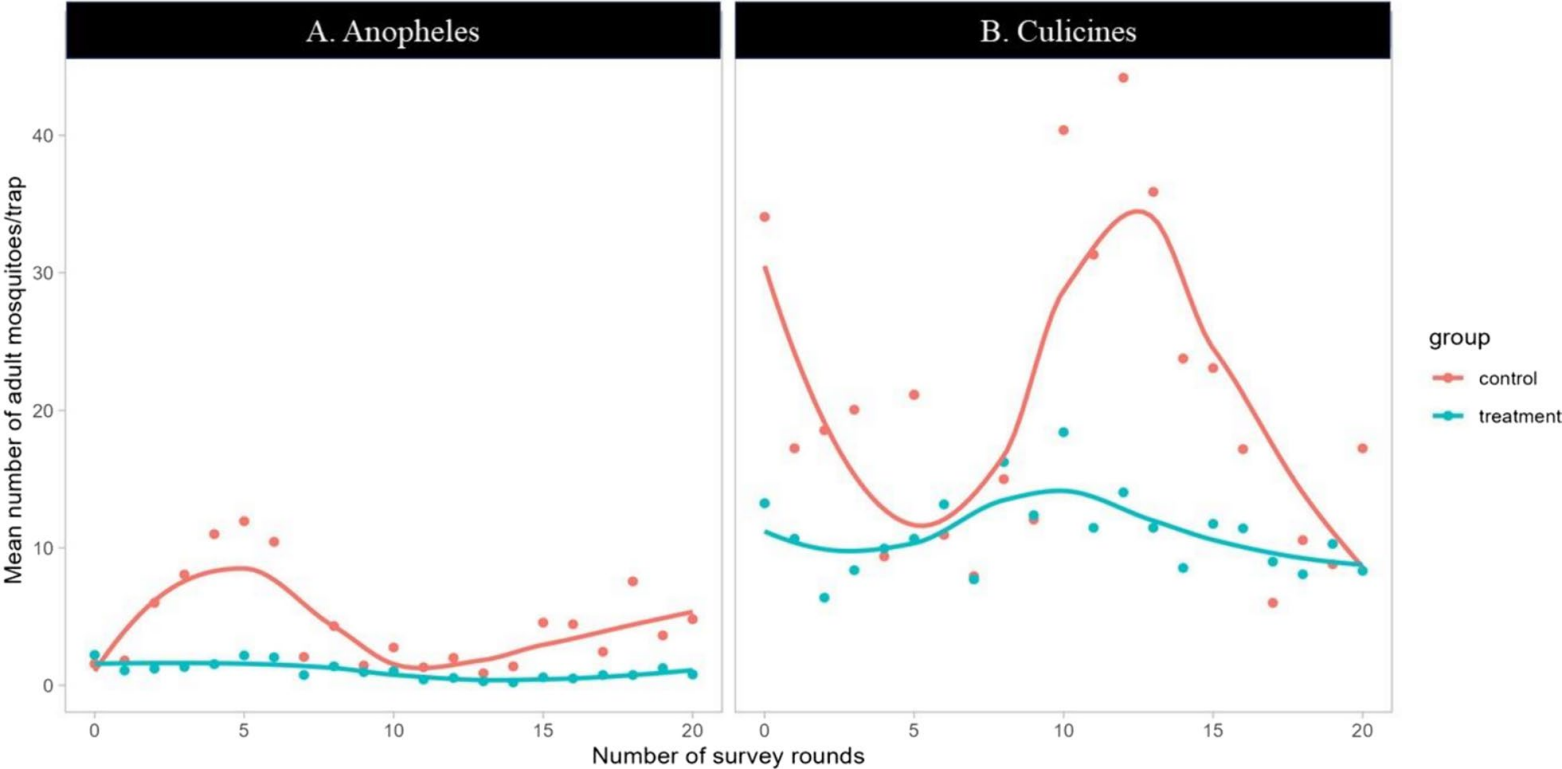
Mean number of adult mosquitoes per house and per survey round collected using CDC-Light trap in treatment and control arms for *Anopheles* spp (**A**), and culicines spp (**B**)

**Fig. 7:**
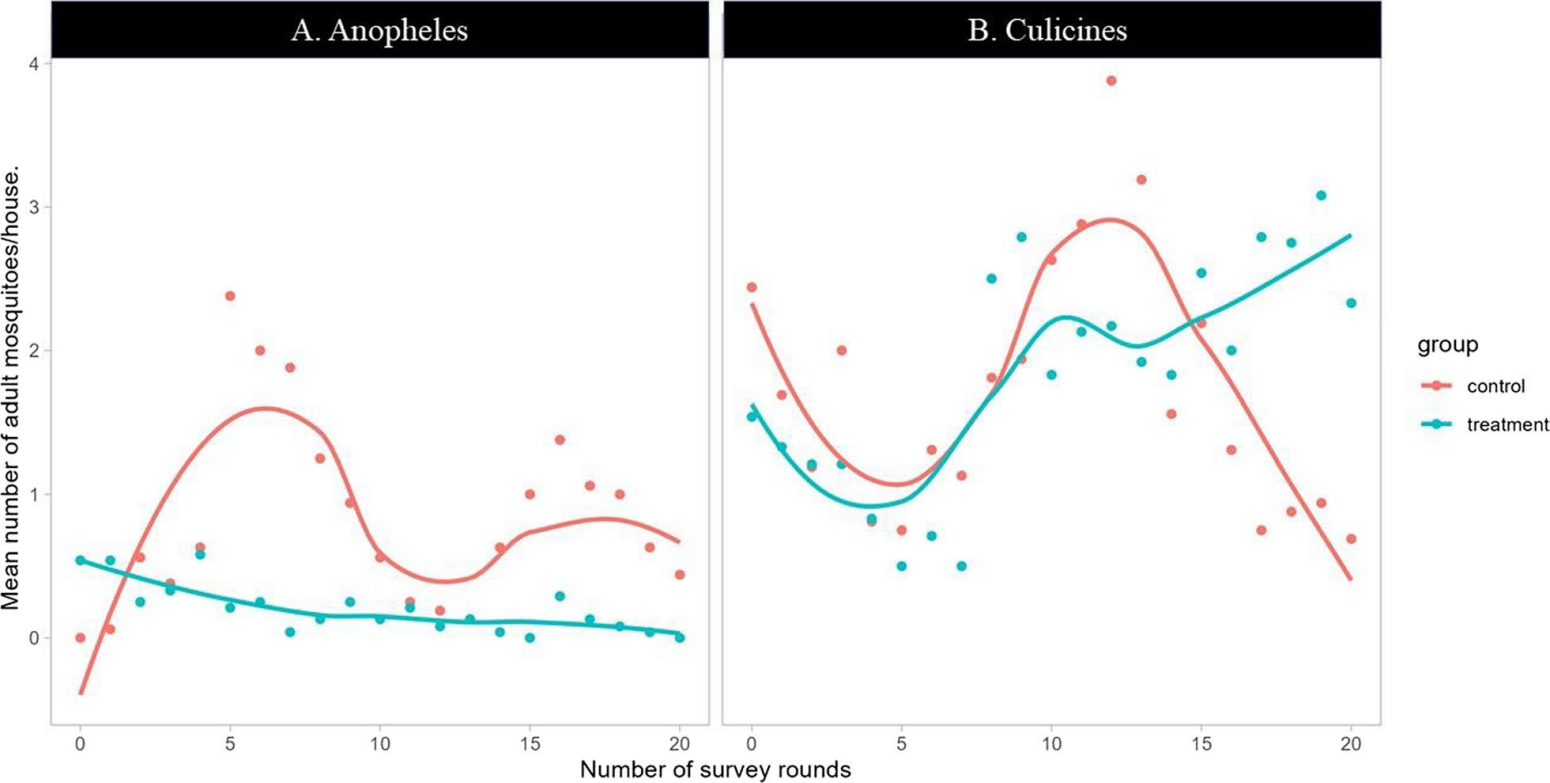
Mean number of adult mosquitoes per house and per survey round collected using pyrethrum spraying catching (PSC) method in nearby villages to the treatment (n = 24 houses) and control (n = 16 houses) arms for *Anopheles* spp (**A**), and culicines spp (**B**)

Following *Bti* application, the catches of adult Anopheles mosquitoes were significantly reduced by 78.7% and by 79.8% for total *Anopheles* and *An. gambiae s.l.,* respectively (Table 7). However, the collections of culicines spp. increased significantly by 10.12% (p < 0.001) in treatment arm with incremental increase from the 5th round (Fig. 6) and a decline trend in control arm (Fig. 7). The two variables of time and treatment alone or in combination did not have a significant effect on adult mosquitoes collected using PSC method, except the combined effects reported on culicine spp. (Table 8).

**Table 7:** Mean number of adult mosquitoes per house and per night collected using Pyrethrum spray catch (PSC) in nearby villages of treatment (n = 480) and control arms (n = 320), during the 20 rounds of drones-based application of bio-larvicide (Bti), Rwanda

Mosquito species	Control arm: Mean + SE ( n = 320)	Treatment arm: Mean + SE (n = 480)	Mean difference + SE	Reduction in %	P-Value
*Total* Anopheles	0.87 (0.08)	0.19 (0.02)	0.68 (0.07)	78.74	< 0.001*
*Culicines spp*	1.68 (0.13)	1.85 (0.10)	− 0.17 (0.17)	− 10.12	< 0.001*
*An. gambiae s.l*	0.84 (0.82)	0.17 (0.02)	0.66 (0.07)	79.76	< 0.001*

The “*” explains the statistical differences at 95% of confidence interval

**Table 8:** Effect of treatment and time alone or in combination on anopheles, culicines adult mosquitoes collected using PSC method

	**Coefficient**	**[95% conf. Interval]**
Anopheles spp.		
Treatment	− 0.39	(− 0.79;0.01)
Time	0.00	(− 0.02;0.02)
Time × Treatment	− 0.02	(− 0.05;0.00)
Culicines spp.		
Treatment	− 0.98	(− 3.76;1.81)
Time	− 0.02	(− 0.06;0.02)
Time × Treatment	0.11***	(0.06;0.16)

### Malaria incidence

At the start of larviciding intervention in July 2020, 72 cases of malaria were reported from the 12 contingent villages to intervention site with an incidence rate of 2.6 cases per 1000 inhabitants for a population of 27,254 people. In April 2021, at the end of the intervention, malaria cases dropped to 35 cases with 131 cases for 1000 inhabitants. However, in control site, 40 and 39 malaria cases were reported at the beginning and at end of the intervention with an incidence rates of 5.60 and 5.53 cases per 1000 inhabitants, respectively. It was found that malaria incidence declined significantly between intervention and control sites (U = 20, z = − 2.268, p = 0.023) with a specific contribution of larviciding estimated to 33.1% to the ITNs distributed in February 2020 in both study sites.

## Discussion

The findings of this LSM trial performed in irrigated rice fields of sub-urban Kigali, Rwanda, using drone-based technology showed a significant reduction (P < 0.001) of mosquito habitat occupancies with a decline of 78.3%, 69.2% and 31.4% for anopheline, culicine larvae and pupae stages, respectively. The mosquito larval density also declined significantly (p < 0.001) with 98.7%, 75.0% and 81.3% for anopheline and culicine larvae and pupae, respectively. The mean number of adult mosquitoes per trap and per night caught with CDC-LT decreased significantly (P < 0.001) with reduction of 79.1% for total *Anopheles* species, 44.1 for culicines and 76.05 for *An. gambiae* s.l. the primary malaria vector in study site. The mean number of mosquitoes caught per house and per night with PSC method also significantly declined (P < 0.001) compared to the control site for total *Anopheles* and *An. gambiae* s.l. with respective reduction of 78.7% and 79.8%. However, the culicine mosquitoes increased significantly by 10.1% (p < 0.001)**.** Malaria incidences reported by community health workers in contingent villages were higher in control than intervention sites (U = 20, z = − 2.268, p = 0.023).

Similarly, another previous larval source management trial conducted for six months in rice fields in southeast Rwanda, with *Bti* solely sprayed with simple knapsack sprayer pumps also showed significant reduction in mosquito habitat occupancies as well as the density of aquatic stages and adult *Anopheles* mosquitoes collected using CDC-LT. This trial reported a complete interruption of pupal stage in treated arm from the 5th round of the 12 rounds of *Bti* application [15]. Moreover, the beneficiary communities, mainly rice farmers, demonstrated a high perception of *Bti* safety and acceptance of the larviciding intervention [27]. Therefore, this study demonstrated the limitations of larviciding using simple knapsack in complex and large mosquito habitats such as irrigated rice fields. The *Bti* sprayers reported the difficulties encountered during the *Bti* application mainly the muddy and slippery soils during rainy seasons, watering of rice plots mainly the first three months of rice farming cycle, and the coverage of upstream water dam for storage of irrigation water [15]. The above challenges were addressed during the current larviciding trial by prior mapping of potential mosquito breeding sites and the aerial spraying of *Bti* using drones instead of simple knapsacks. This trial yielded a high entomological impact compared to the previous trial using hand knapsack sprayer pumps.

The moderate impact of larviciding with Bti (VectoBac WDG) was also documented by prior studies in complex mosquito breeding habitats such as flooded ecosystem in Gambia [14], and irrigated rice fields [10]. Another targeted larviciding trial with only treatment of the most productive *Anopheles* breeding sites and systematic treatment of all potential mosquito breeding areas demonstrated similar reduction of female anopheles mosquitoes respectively by 61% and 70% [28]. The larviciding trial with VectorMax® G conducted in Yaoundé, Cameroon found an impact both on malaria vectors and *Culex* mosquitoes with reduction of 69% and 36.6% in aquatic habitats and adult density inside houses for *Culex* species, respectively [29]. The impact on other mosquito species such as *Aedes* spp. and *Culex* mosquitoes was also found to be limited in some settings [30]. The timing of larviciding, exhaustive and accurate geo-locations and types of mosquito habitats were the primary hindrances to optimize the impact of LSM, and thus requiring the new technologies [16, 31]. The other limitations associated to the impact of larviciding are the accurate detection of water bodies potential to mosquito breeding areas, the human resettlements and nature of constructions. These are to-date sorted out by prior mapping of the study areas with drones and enabling then the improvement of the coverage of mosquito larval habitats with larviciding [32].

The recent trial of drones based larviciding carried out in Unguja island, Zanzibar, Tanzania in rice fields showed an improved entomological impact of more than 90% reduction of aquatic stages abundance and density of mosquitoes. This study demonstrated that Unmanned Aerial Vehicles (UAVs) improved the coverage of larviciding and can be used beyond the targeted sites as recommended by the WHO for LSM intervention [12]. The UAVs technologies proved the effectiveness of controlling mosquito population over large and complex areas at low cost. They may also enhance the cost-effective control of malaria and other mosquito borne diseases and elimination efforts [33]. Another promising technology is based on high resolution images captured with drones which may also contribute to geo-locating at low costs the potential mosquito larval habitats. The latter are detected through the analysis of presence and type of aquatic vegetation [34]. Therefore, the required technical skills [35] and the more time for processing of images still the main hindrances for this innovation which guide to tackle directly the positive larval mosquito breeding habitats during larviciding operation [34]. The spatial intelligence system (SIS) for larviciding is another promising technology and based on mapping of mosquito breeding areas which was shown cheaper and more accurate than the conventional ground-based mapping method [16].

The larviciding conducted in many mosquito habitats including the complex ecosystems, with improved coverage and appropriate larvicide product demonstrated a drastic impact on abundance and density of malaria vectors and other mosquito borne diseases. Nowadays, larviciding is a proven and viable intervention enabling to supplement the current core indoor vector control tools in targeted settings even in large and complex mosquito breeding habitats [28, 29, 33, 36, 37]

## Conclusions

Despite the targeted study area was a complex ecosystem of irrigated rice fields with frequent flooded areas and man-made breeding sites, the Unmanned aerial vehicles (drones) contributed to operationalize the larviciding intervention which was guided by prior aerial maps of water bodies. The coverage of all geo-located potential breeding sites was effectively performed alongside the 20 rounds of larviciding operations. In comparison between the intervention and control site, the aquatic mosquito larval habitat occupancies and mosquito larval density were significantly reduced, as well as the adult *Anopheles* collected using CDC-LT and PSC methods. The malaria incidence showed also a significant decline in intervention compared to the control site. The scaling up of larviciding using drone technologies for prior mapping of potential mosquito breeding sites and then spraying of larvicide proved its effectiveness not only in targeted sites as recommended by the WHO but also in complex *Anopheles* breeding habitats. These vector control technologies have to be integrated by malaria programs to enhance the gains in malaria control and elimination efforts. However, the low impact on culicine mosquitoes may affect the acceptance of LSM intervention by beneficiary communities. Further engagement of local communities is recommended and using appropriate formulations of larvicide for controlling culicines breeding habitats.

## Data Availability

No datasets were generated or analysed during the current study.

## Abbreviations

*Bti*: *Bacillus thuringiensis* Var. *israelensis*
CDC: Centers for Disease Control and Prevention
CTAB: Cetyl Trimethyl Ammonium Bromide
ELISA: Enzyme-Linked Immunosorbent Assay
IRS: Indoor residual spraying
ITU: International toxic units
LLIN: Long-lasting insecticide treated net
LSM: Larval source management
MOH: Ministry of Health
PCR: Polymerase chain reaction
PSC: Pyrethrum spraying collection
UAV: Unmanned aerial vehicles
WDG: Water dispersible granules
WHO: World Health Organization

## References

[1] Mbewe RB, Keven JB, Mzilahowa T, Mathanga D, Wilson M, Cohee L, et al. Blood-feeding patterns of *Anopheles* vectors of human malaria in Malawi: implications for malaria transmission and effectiveness of LLIN interventions. Malar J. 2022;21:67. 35241083 10.1186/s12936-022-04089-7 PMC8892392

[2] Siya A, Kalule BJ, Ssentongo B, Lukwa AT, Egeru A. Malaria patterns across altitudinal zones of Mount Elgon following intensified control and prevention programs in Uganda. BMC Infect Dis. 2020;20:425. 32552870 10.1186/s12879-020-05158-5 PMC7301530

[3] WHO. World malaria report 2022. Geneva: World Health Organization; 2023.

[4] Wiebe A, Longbottom J, Gleave K, Shearer FM, Sinka ME, Massey NC, et al. Geographical distributions of African malaria vector sibling species and evidence for insecticide resistance. Malar J. 2017;16:85. 28219387 10.1186/s12936-017-1734-y PMC5319841

[5] MOH-Rwanda. 2016_Annual_Statistical_booklets, vol. 9. Kigali: Ministry of Health; 2018.

[6] Karema C, Wen S, Sidibe A, Smith JL, Gosling R, Hakizimana E, et al. History of malaria control in Rwanda: implications for future elimination in Rwanda and other malaria-endemic countries. Malar J. 2020;19:356. 33028337 10.1186/s12936-020-03407-1 PMC7539391

[7] Animut A, Negash Y. Dry season occurrence of *Anopheles* mosquitoes and implications in Jabi Tehnan District, West Gojjam Zone, Ethiopia. Malar J. 2018;17:445. 30497495 10.1186/s12936-018-2599-4 PMC6267885

[8] Vigodny A, Ben Aharon M, Wharton-Smith A, Fialkoff Y, Houri-Yafin A, Bragança F, et al. Digitally managed larviciding as a cost-effective intervention for urban malaria: operational lessons from a pilot in São Tomé and Príncipe guided by the Zzapp system. Malar J. 2023;22:114. 37024950 10.1186/s12936-023-04543-0 PMC10080920

[9] Hemingway J, Shretta R, Wells TNC, Bell D, Djimdé AA, Achee N, et al. Tools and strategies for malaria control and elimination: what do we need to achieve a grand convergence in malaria? PLoS Biol. 2016;14: e1002380. 26934361 10.1371/journal.pbio.1002380 PMC4774904

[10] Fillinger U, Lindsay SW. Larval source management for malaria control in Africa: myths and reality. Malar J. 2011;10:353. 22166144 10.1186/1475-2875-10-353 PMC3273449

[11] Killeen GF, Tatarsky A, Diabate A, Chaccour CJ, Marshall JM, Okumu FO, et al. Developing an expanded vector control toolbox for malaria elimination. BMJ Glob Health. 2017;2: e000211. 28589022 10.1136/bmjgh-2016-000211 PMC5444090

[12] WHO. Larval source management—a supplementary measure for malaria vector control. An operational manual. Geneva: World Health Organization; 2013.

[13] Maheu-Giroux M, Castro MC. Impact of community-based larviciding on the prevalence of malaria infection in Dar es Salaam, Tanzania. PLoS One. 2013;8: e71638. 23977099 10.1371/journal.pone.0071638 PMC3743749

[14] Majambere S, Pinder M, Fillinger U, Ameh D, Conway DJ, Green C, et al. Is mosquito larval source management appropriate for reducing malaria in areas of extensive flooding in the Gambia? A cross-over intervention trial. Am J Trop Med Hyg. 2010;82:176–84. 20133989 10.4269/ajtmh.2010.09-0373 PMC2813154

[15] HakizimanaIngabire ECM, Rulisa A, Kateera F, Van de Borne B, Muvunyi CM, et al. Community-based control of malaria vectors using *Bacillus**thuringiensis* var. israelensis (Bti) in Rwanda. Int J Environ Res Public Health. 2022;19:6699. 35682283 10.3390/ijerph19116699 PMC9180564

[16] Hardy A, Haji K, Abbas F, Hassan J, Ali A, Yussuf Y, et al. Cost and quality of operational larviciding using drones and smartphone technology. Malar J. 2023;22:286. 37759213 10.1186/s12936-023-04713-0 PMC10523724

[17] National Institute of Statistics of Rwanda, Rwanda. Ministry of Health, MEASURE DHS (Program). Rwanda demographic and health survey, 2014–15: final report. 2016.

[18] Muhire I, Ahmed F, Abutaleb K. Spatio-temporal variations of rainfall erosivity in Rwanda. J Soil Sci Environ Manag. 2015;6:72–83.

[19] Gasabo district. Gasabo district potentialities assessment for the integrated and self-centered local. Kigali; 2013.

[20] WHO. WHOVC-SP_Bti_strain_AM65–52+Bsph_strain_ABTS-1743_2016. Geneva: World Health Organization; 2016.

[21] Mboera LEG, Kihonda J, Braks MAH, Knols BGJ. Influence of centers for disease control light trap position, relative to a human-baited bed net, on catches of *Anopheles gambiae* and *Culex quinquefasciatus* in Tanzania. Am J Trop Med Hyg. 1998;59:595–6. 9790436 10.4269/ajtmh.1998.59.595

[22] Onyango SA, Kitron U, Mungai P, Muchiri EM, Kokwaro E, King CH, et al. Monitoring malaria vector control interventions: effectiveness of five different adult mosquito sampling methods. J Med Entomol. 2013;50:1140–51. 24180120 10.1603/me12206 PMC3975164

[23] Coetzee M. Key to the females of Afrotropical *Anopheles* mosquitoes (Diptera: Culicidae). Malar J. 2020;19:70. 32054502 10.1186/s12936-020-3144-9 PMC7020601

[24] Scott JA, Brogdon WG, Collins FH. Identification of single specimens of the *Anopheles gambiae* complex by the polymerase chain reaction. Am J Trop Med Hyg. 1993;49:520–9. 8214283 10.4269/ajtmh.1993.49.520

[25] Appawu MA, Bosompem KM, Dadzie S, McKakpo US, Anim-Baidoo I, Dykstra E, et al. Detection of malaria sporozoites by standard ELISA and VecTest™ dipstick assay in field-collected anopheline mosquitoes from a malaria endemic site in Ghana. Trop Med Int Health. 2003;8:1012–7. 14629768 10.1046/j.1360-2276.2003.00127.x

[26] Getachew D, Gebre-Michael T, Balkew M, Tekie H. Species composition, blood meal hosts and *Plasmodium* infection rates of *Anopheles* mosquitoes in Ghibe River Basin, southwestern Ethiopia. Parasit Vectors. 2019;12:257. 31122286 10.1186/s13071-019-3499-3 PMC6533711

[27] Ingabire CM, Hakizimana E, Rulisa A, Kateera F, Van de Borne B, Muvunyi CM, et al. Community-based biological control of malaria mosquitoes using *Bacillus**thuringiensis* var. israelensis (Bti) in Rwanda: community awareness, acceptance and participation. Malar J. 2017;16:399. 28974204 10.1186/s12936-017-2046-y PMC5627396

[28] Dambach P, Baernighausen T, Traoré I, et al. Reduction of malaria vector mosquitoes in a large-scale intervention trial in rural Burkina Faso using Bti based larval source management. Malar J. 2019;18:311. 31521176 10.1186/s12936-019-2951-3 PMC6744650

[29] Talipouo A, Doumbe-Belisse P, Ngadjeu CS, Djamouko-Djonkam L, Nchoutpouen E, Bamou R, et al. Larviciding intervention targeting malaria vectors also affects *Culex* mosquito distribution in the city of Yaoundé, Cameroon. Curr Res Parasitol Vector-Borne Dis. 2023;4: 100136. 37693015 10.1016/j.crpvbd.2023.100136 PMC10491826

[30] Dambach P, Bärnighausen T, Yadouleton A, Dambach M, Traoré I, Korir P, et al. Is biological larviciding against malaria a starting point for integrated multi-disease control? Observations from a cluster randomized trial in rural Burkina Faso. PLoS ONE. 2021;16: e0253597. 34143831 10.1371/journal.pone.0253597 PMC8213177

[31] Hardy A, Makame M, Cross D, Majambere S, Msellem M. Using low-cost drones to map malaria vector habitats. Parasit Vectors. 2017;10:29. 28088225 10.1186/s13071-017-1973-3 PMC5237572

[32] Carrasco-Escobar G, Manrique E, Ruiz-Cabrejos J, Saavedra M, Alava F, Bickersmith S, et al. High-accuracy detection of malaria vector larval habitats using drone-based multispectral imagery. PLoS Negl Trop Dis. 2019;13: e0007105. 30653491 10.1371/journal.pntd.0007105 PMC6353212

[33] Mukabana WR, Welter G, Ohr P, Tingitana L, Makame MH, Ali AS, et al. Drones for area-wide larval source management of malaria mosquitoes. Drones. 2022;6:180.

[34] Stanton MC, Kalonde P, Zembere K, Hoek Spaans R, Jones CM. The application of drones for mosquito larval habitat identification in rural environments: a practical approach for malaria control? Malar J. 2021;20:244. 34059053 10.1186/s12936-021-03759-2 PMC8165685

[35] Derua YA, Kweka EJ, Kisinza WN, Githeko AK, Mosha FW. Bacterial larvicides used for malaria vector control in sub-Saharan Africa: review of their effectiveness and operational feasibility. Parasit Vectors. 2019;12:426. 31470885 10.1186/s13071-019-3683-5 PMC6716942

[36] Olalubi OA, Chinwe GK. Promoting larval source management as a vital supplemental addendum and more likely cost-effective approach for malaria vector control in Nigeria. Prev Infect Control. 2016;2:2.

[37] Mpofu M, Becker P, Mudambo K, De Jager C. Field effectiveness of microbial larvicides on mosquito larvae in malaria areas of Botswana and Zimbabwe. Malar J. 2016;15:586. 27923385 10.1186/s12936-016-1642-6 PMC5139019

